# Individual patient data meta-analysis of dynamic cerebral autoregulation and functional outcome after ischemic stroke

**DOI:** 10.1161/STROKEAHA.123.045700

**Published:** 2024-05

**Authors:** Lucy Beishon, Terrie Vasilopoulos, Angela SM Salinet, Brooke Levis, Samuel Barnes, Eleanor Hills, Pranav Ramesh, Panagoula Gkargkoula, Jatinder S. Minhas, Pedro Castro, Patrice Brassard, Nicolai Goettel, Erik D. Gommer, Jose Luis Jara, Jia Liu, Martin Mueller, Nathalie Nasr, Stephen Payne, Andrew D. Robertson, David Simpson, Thompson G Robinson, Ronney B. Panerai, Ricardo C. Nogueira

**Affiliations:** a University of Leicester, Department of Cardiovascular Sciences, Leicester, UK; b NIHR Leicester Biomedical Research Centre, British Heart Foundation Cardiovascular Research Centre, Glenfield Hospital, Leicester, UK; c Department of Anesthesiology, University of Florida College of Medicine, Gainesville, FL, USA; d Neurology Department, Hospital das Clinicas, School of Medicine, University of Sao Paulo, Sao Paulo, Brazil; e Lady Davis Institute for Medical Research, Jewish General Hospital, Montreal, Quebec, Canada; f Centre for Prognosis Research, School of Medicine, Keele University, Staffordshire, UK; g University Hospitals of Leicester NHS Trust, Leicester, UK; h Department of Neurology, Centro Hospitalar Universitário de São João, Faculty of Medicine, University of Porto; i Département de Kinésiologie, Faculté de médecine, Institut universitaire de cardiologie et de pneumologie de Québec; j Department of Anesthesiology, Perioperative and Pain Medicine, Brigham and Women’s Hospital, Harvard Medical School, Boston, MA, USA; k Department of Clinical Neurophysiology, Maastricht University Medical Centre, Maastricht, the Netherlands; l Departamento de Ingeniería Informática, Universidad de Santiago de Chile; m Shenzhen Institutes of Advanced Technology at the Chinese Academy of Sciences in Shenzhen, China; n Department of Neurology and Neurorehabilitation, Spitalstrasse, CH 6000 Lucerne; o Department of Neurology, Poitiers University Hospital, Laboratoire de Neurosciences Expérimentales et Cliniques, University of Poitiers, France; p Institute of Applied Mechanics, National Taiwan University, Taipei, Taiwan; q Schlegel-UW Research Institute for Aging, University of Waterloo, Waterloo, ON, CA; r Faculty of Engineering and Physical Sciences, University of Southampton

**Keywords:** Acute stroke, cerebral ischemia, cerebrovascular autoregulation, transcranial Doppler ultrasonography, meta-analysis, IPDMA

## Abstract

**Background:**

The relationship between dynamic cerebral autoregulation (dCA) and functional outcome after acute ischemic stroke (AIS) is unclear. Previous studies are limited by small sample sizes and heterogeneity.

**Methods:**

We performed a one–stage individual patient data meta–analysis to investigate associations between dCA and functional outcome after AIS. Participating centers were identified through a systematic search of the literature and direct invitation. We included centers with dCA data within one year of AIS in adults aged over 18 years, excluding intracerebral or subarachnoid hemorrhage. Data were obtained on phase, gain, coherence, and autoregulation index derived from transfer function analysis at low frequency (LF) and very low frequency (VLF) bands. Cerebral blood velocity (CBv), arterial pressure, end-tidal carbon dioxide, heart rate, stroke severity and sub-type, and comorbidities were collected where available. Data were grouped into four time points after AIS: <24 hours (h), 24–72h, 4–7 days (d), and >3 months (mo). The modified Rankin scale (mRS) assessed functional outcome at 3mo. mRS was analyzed as both dichotomized (0-2 vs. 3-6) and ordinal (mRS: 0–6) outcomes. Univariable and multivariable analyses were conducted to identify significant relationships between dCA parameters, comorbidities, and outcomes, for each time point using generalized linear (dichotomized outcome), or cumulative link (ordinal outcome) mixed models. The participating center was modeled as a random intercept to generate odds ratios (OR) with 95% confidence intervals (CI).

**Results:**

The sample included 384 individuals (35% female) from seven centers, aged 66.3±13.7 years, with predominantly non-lacunar stroke (n=348, 69%). In the affected hemisphere, higher phase at VLF predicted better outcome (dichotomized mRS) at <24h (crude OR=2.17, 95% CI:1.47-3.19, p<0.001), 24–72h (crude OR=1.95, 95% CI:1.21-3.13, p=0.006), and phase at LF predicted outcome at 3mo (crude OR=3.03, 95%CI:1.10–8.33, p=0.032). These results remained after covariate adjustment.

**Conclusions:**

Greater transfer function analysis-derived phase was associated with improved functional outcome at 3mo after AIS. dCA parameters in the early phase of AIS may help to predict functional outcome.

## Graphical abstract

**Figure:**
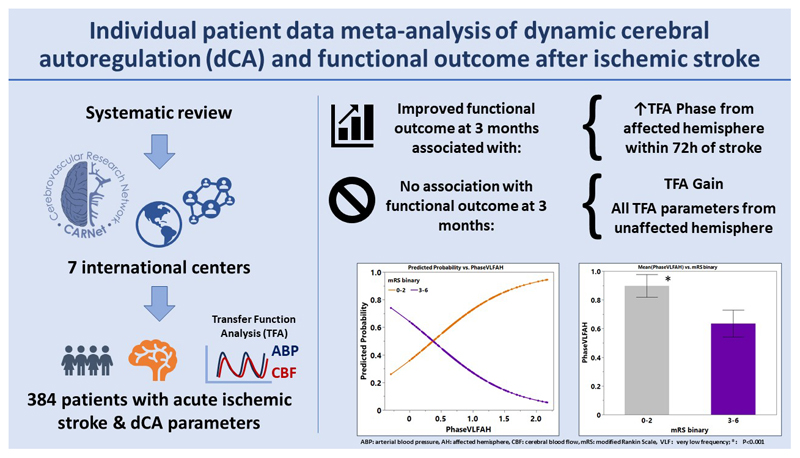
Graphical abstract

## Non-Standard Abbreviations and Acronyms

AIS: Acute ischaemic stroke
ARI: Autoregulation index
BP: Blood pressure
CAI: Cerebral augmentation index
CARNet: Cerebrovascular Research Network
CBv: Cerebral blood velocity
CI: Confidence interval
CLMM: Cumulative link mixed models
dCA: Dynamic cerebral autoregulation
EtCO_2_: End-tidal CO_2_
GLMM: Generalized linear mixed models
HR: Heart rate
IPDMA: Individual patient data meta-analysis
LF: Low frequency
Mo: Months
mRS: Modified Rankin Scale
NIHSS: National Institute for Health Stroke Scale
OR: Odds ratio
PP: Predictive probabilities
STROBE: Strengthening the Reporting of Observational Studies in Epidemiology
TCD: Transcranial Doppler ultrasonography
TFA: Transfer function analysis
VLF: Very low frequency

## Introduction

**Figure 1:**
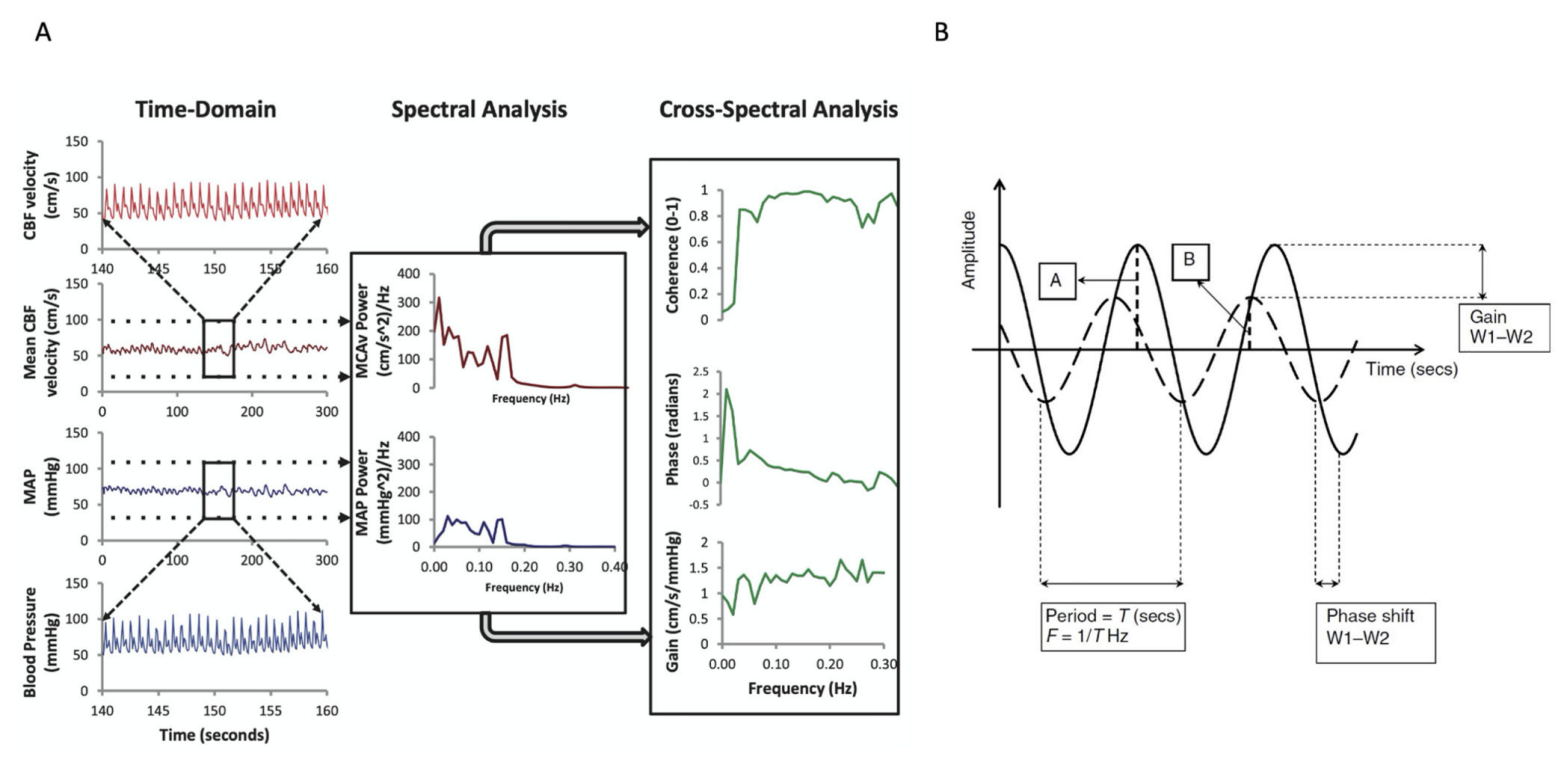
Dynamic Cerebral Autoregulation (dCA) determined via transfer function analysis (TFA). Panel A illustrates the conversion of cerebral blood velocity (CBv) and mean arterial pressure (MAP) from the time to frequency domain to derive metrics of coherence, gain and phase. Panel B illustrates the phase shift (A) between the blood pressure and CBv waveforms, and the difference in amplitude (gain, B) between the blood pressure and CBv waveforms. MCAv=middle cerebral artery blood velocity. Figure adapted from^8,9^ with permission.

Dynamic cerebral autoregulation (dCA) is a continuous, active process, to maintain appropriate cerebral perfusion through regulation of the cerebral vasculature in the face of surrounding insults^1^. One potential insult to the cerebral tissue is ischemia in the form of an acute ischemic stroke (AIS). Impaired dCA in this context has been associated with greater stroke severity and clinical deterioration^2,3^. To date, studies have demonstrated that dCA is altered in the affected hemisphere of severe AIS^2,4–6^. dCA is a potential, non-invasive biomarker that can be measured at the bedside, and maybe able to prognosticate outcome in the early stages following AIS^1^. In turn, this could guide clinical practice in the acute stage, particularly given recent advancements in acute reperfusion therapies (thrombolysis and thrombectomy). However, there remains a lack of large, adequately powered studies investigating the association between dCA and functional outcome following AIS. Individual patient data meta-analysis (IPDMA) can overcome these limitations, by minimizing inherent heterogeneity at study level, improving the standardization of analyses and reliability of pooled estimates^7^. dCA evaluates the influence of oscillations in blood pressure (BP) exerted on cerebral blood flow. Often, cerebral blood velocity (CBv), measured by transcranial Doppler ultrasonography (TCD), is used as a surrogate of flow (Figure 1)^1,8,9^. Although there exists several analytical methods to quantify dCA, transfer function analysis (TFA) represents a popular approach, which quantifies the extent to which fluctuations in BP are buffered by the cerebrovasculature and thus transmitted to CBv^1^. Fluctuations in BP (e.g. thigh-cuff maneuver or repeated squat-stands), or from spontaneous beat-to-beat changes in BP that occur more naturally^1^. In AIS, spontaneous fluctuations are used more commonly due to practicality and patient tolerability. TFA provides three main metrics of dCA: 1) coherence, describing the relative amount of output power that is explained by the input at each given frequency; 2) gain, describing the dampening of BP changes on CBv (higher gain reflects reduced dCA efficiency); and 3) phase, describing the shift between BP and CBv waveforms (higher phase represents greater dCA efficiency)^1,10^. Other metrics used to evaluate dCA are the autoregulation index (ARI), which is a TFA-derived scale of autoregulatory efficiency from low (0) to high (9)^1^ and cerebral augmentation index (CAI), which describes the percentage increase in CBv relative to baseline^11^.

Despite consensus guidelines on minimum standards for dCA research, heterogeneity remains a significant challenge^10^, such as lack of end-tidal carbon dioxide (EtCO_2_) monitoring, and inclusion of important baseline covariates^7,12^.

Therefore, we conducted a one-stage IPDMA representing the largest amalgamation of dCA data in AIS to date, through the international Cerebrovascular Research Network (CARNet). The primary aim of this analysis was to explore the relationship between dCA and functional outcome following AIS. Secondary objectives were to analyze temporal changes in dCA occurring at the acute (<24 hours [h] and 24–72h), subacute (4–7 days [d]), and chronic (>3 months [mo]) stages after AIS.

## Methods

This study is reported in line with the Preferred Reporting Items for Systematic Reviews and Meta-analyses of IPDMA (Supplementary Material). Data are available on request from the INFOMATAS committee.

### Study design

INFOMATAS is an international, multi-center initiative to investigate the relationship between dCA and outcome after AIS. Participating centers were identified via CARNet, through systematic review (Figure S1) and contacting the authors of relevant articles. Anonymized data were collected from each center. Ethical approval was granted by the University of Leicester ethics committee to collect and analyze anonymous research data for the project (28895-lb330-ls:cardiovascularsciences). The study protocol for this IPDMA was prespecified and published^7^. Detailed methods of the systematic review and protocol changes are described in the Supplementary Methods (SM).

### Data collection

Centers were included if they had indices of dCA, within 12 months of AIS (all sub-types), in adults aged ≥18 years. Studies including patients suffering from hemorrhagic stroke and subarachnoid hemorrhage were excluded. A data sharing agreement was signed by representatives from the INFOMATAS committee (LB and RN) and each participating center. A data dictionary was produced to standardize data terminology and consistency between centers (SM). Data were received by RN and AS who uploaded the data into REDCap (Vanderbilt University, Nashville, TN, USA), a secure web-based application for managing online databases. Data were checked for accuracy, completion, and integrity during this process. Data queries were clarified with the participating center prior to uploading. During data entry, each subject was given a subject identifier, coded by the center of origin to allow for data clustering in the analysis. The final database was checked for accuracy and consistency prior to analysis. Phase was converted to radians (rad; degrees x π/180), when necessary, to harmonize data. Units of gain were cm·s^-1^·mmHg^-1^. A full list of variables in the shared dataset is included in the data dictionary (SM). Quality of data collection and reporting methods were assessed using the CARNet White Paper^10^ and Strengthening the Reporting of Observational Studies in Epidemiology (STROBE) criteria^13^, respectively.

### Data analysis

This was a one-stage IPDMA in which only individual patient data were modelled and analyzed; we did not collect study level or aggregate data for meta-analysis. The primary outcome was functional status measured by the modified Rankin Scale (mRS)^4^. Continuous measures were summarized as means and standard deviation, and categorical measures were summarized as counts and percentages. For the primary analysis, separate analyses were performed with mRS treated as a dichotomized variable (good [mRS:0–2] vs poor [mRS:3–6] outcome) and as an ordinal variable (mRS: 0–6). The association between each dCA parameter and mRS was evaluated using generalized linear mixed models (GLMM) for dichotmized outcomes and cumulative link mixed models (CLMM) for ordinal outcomes. GLMM and CLMM included the study center as a random effect. Effects were summarized as beta estimates with standard error and 95% confidence intervals (CI), as well as odds ratios (OR) with 95% CI (for predictors with means <1, calculations are per unit standard deviation change). CLMMs were only run at <24h and 24–72h due to sample size restrictions. Additionally, predicted probabilities (PP) of having a given mRS score across different levels of dCA parameters were estimated. Both univariable (dCA parameters only) and multivariable models were analyzed. Covariates included in multivariable models were initial stroke severity (National Institutes of Health Stroke Scale; NIHSS), age, non–lacunar/lacunar stroke, diabetes mellitus, and antihypertensive medication use. For smaller samples, only the initial NIHSS score and age were included. All participants were included where data were present for one variable on the univariable analyses. On multivariable analyses, only participants with complete data were included. Comparisons across time points were performed with linear (continuous measures) and generalized linear (categorical) mixed models, with time point as a repeated measure; this would account for the subset of participants who had measurements during multiple time points (<24h, 24-72h, 4-7d, 3mo). These models included a random intercept for center, which means the model allowed the intercept to vary by center. Secondary analyses with NIHSS or stroke type as dependent variable were evaluated. P<0.05 was considered statistically significant. Analyses were performed in R version 4.2.1^14^.

## Results

### Summary of included centers

Seven centers (UK, Portugal, Brazil, Switzerland, China, Taiwan, and Canada) provided data on a total of 384 individuals. Data were derived from 11 studies^2,11,15–23^ and further unpublished data were provided by authors MM and ADR (Table 1). A summary of data provided by each center is shown in Table S1. Most studies enrolled participants prospectively within the acute stage of stroke, and measurements were conducted at multiple time points (<24h to 6mo). In the original studies, six measured the ARI, four provided estimates of phase and gain, and one reported CAI. Alongside TCD, all studies measured beat-to-beat BP (mean arterial pressure); although only eight studies measured BP, heart rate [HR], and EtCO_2_, as recommended by TFA guidelines^10^. Four studies reported mRS at 3mo. Three studies did not report functional outcomes (unpublished data were requested from authors where available). Two studies evaluated radiological outcomes (infarct volumes, cerebral edema, hemorrhagic transformation), in addition to clinical outcomes. Most studies demonstrated a reduction in dCA at varying time points after AIS, but not all evaluated the relationship with clinical outcomes.

### Reporting quality

The median STROBE criteria (Table S2) score was 17/22 points (interquartile range: 16.0–17.5) across the included studies. Areas of poor reporting included: description of the study design, setting, location and recruitment, sample size calculations, attempts to address sources of bias, and description of the participant flow and dropouts. However, the remaining items were generally well reported across the included studies. A summary of the reporting quality for each primary research study are shown in Table S3.

### Summary of heterogeneity and quality of data collection methods (CARNet white paper criteria)

Many details were not consistently described in the original articles; however, it is unclear if these were performed but not reported, and some may not be possible in the AIS population (e.g., abstention from alcohol, nicotine, or vigorous exercise in hospitalized patients). Other criteria may be difficult to control in clinical studies, such as time of day, noise, and environment. In the methods, body position and resting period prior to assessment were the most commonly reported factors, as were the control of sensory stimuli and the environmental conditions. Medications were rarely reported. All studies used TCD, but not all reported EtCO_2_, and none reported intracranial pressure measurements. All studies used a minimum recording duration of five minutes. Most used beat-to-beat data and visually inspected the results, but more technical details (such as filtering and detrending) were rarely mentioned. A considerable number did not report TFA methods, so compliance with White Paper recommendations^10^ were difficult to assess, although the signal processing prior to TFA is still relevant in these studies.

### Demographics and clinical characteristics

A summary of the demographics (overall population and by time period), and mRS scores are provided in Tables S4,5. A total of 384 patients (483 data points) were included in the final analysis based on complete data for the association with mRS at 3mo. The mean age of participants was 65.4±13.8 years; the majority were male (n=261, 67%) with non-lacunar stroke (n=235, 61%). Mean stroke severity (NIHSS) was 8.4±6.8, and a high proportion had arterial hypertension (n=213, 59%) and were on statin therapy (n=148, 53%).

### Outcome analyses

#### dCA variables and outcome

##### Affected hemisphere

**Figure 2:**
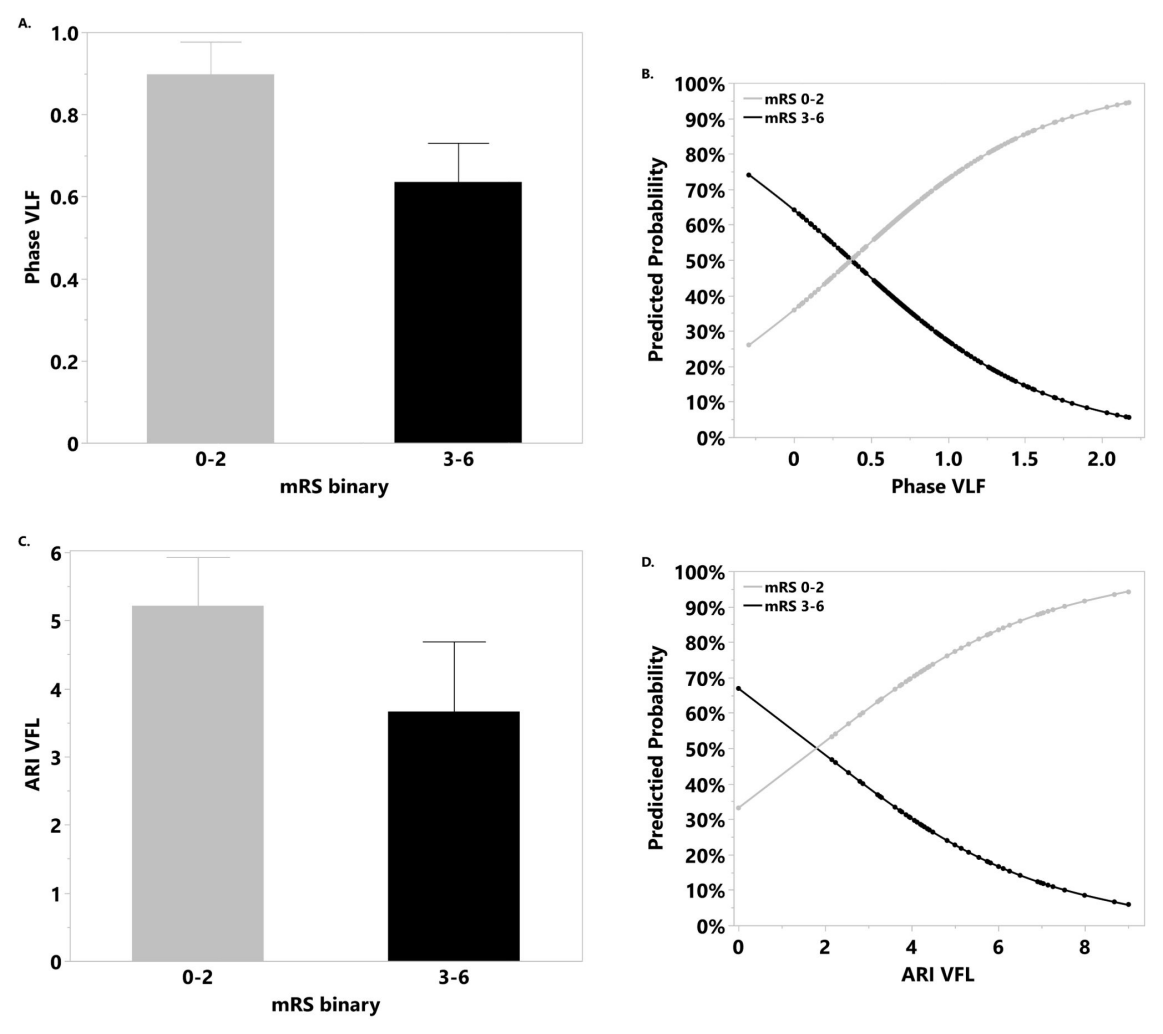
Mean phase at very low frequency (VLF) (A), autoregulation index (ARI) (C) in the affected hemisphere (AH) within 24h in participants with good (modified Rankin Scale [mRS]:0–2) vs poor [mRS 3–6]) outcome at 3mo. The predicted probability of good vs poor outcome with increasing phase at VLF (B), and ARI (D).

Table 2 shows the univariable results for the relationship of dCA with mRS at 3mo. Higher VLF phase at <24h was predictive of improved outcome for dichotomized mRS (Table 2, Figure 2A). For dichotomized mRS, the probability of better outcome (mRS:0–2) increased as VLF phase increased (Figure 2B). In contrast, the probability of mRS≥3 was inversely associated with VLF phase. This association remained significant after covariate adjustment (OR=2.29, 95% CI:1.44-3.64, p<0.001).

Higher ARI in the affected hemisphere was predictive of better outcome (Table 2; Figure 2C), However, this was non-significant following covariate adjustment (OR=1.33, 95% CI:0.55-3.24, p=0.18).

**Figure 3:**
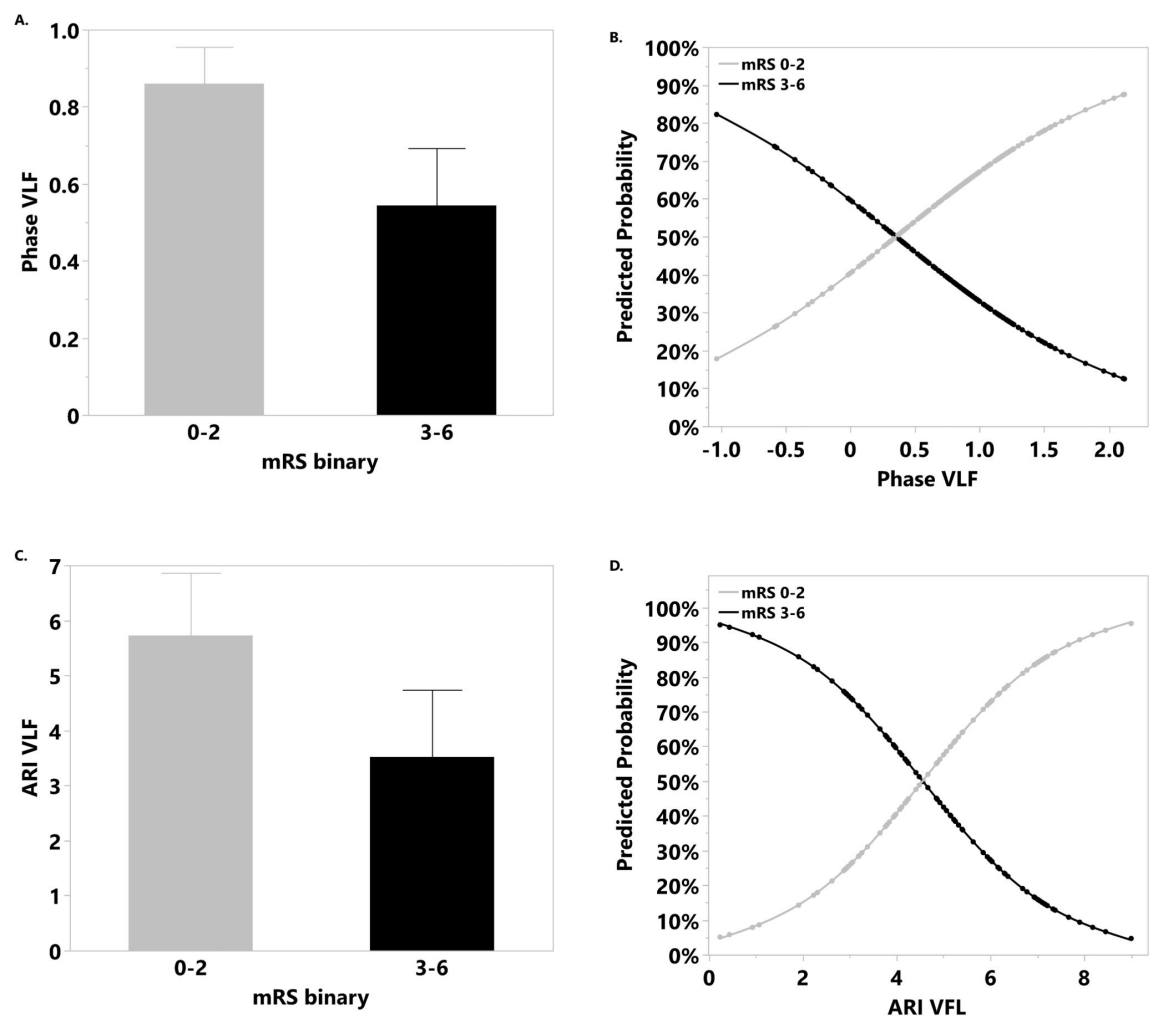
Mean phase at very low frequency (VLF) (A), autoregulation index (ARI) (C) in the affected hemisphere (AH) at 24–72h in participants with good (modified Rankin Scale [mRS]:0–2) vs poor (mRS:3–6) outcome at 3mo. The predicted probability of good vs poor outcome with increasing phase at VLF (B), and ARI (D).

At 24–72h, univariate associations with dichotomized mRS for VLF phase and ARI were maintained (Table 2 and Figure 3), in addition to LF phase, LF gain, and CBv (Table 2). Following covariate adjustment, associations remained for VLF phase (OR=2.5, 95% CI:1.14-5.47, p=0.023) and LF phase (OR=2.58, 95% CI:1.25-5.32, p=0.01). However, there was no association between dCA at 4-7d and outcome. Finally, at 3mo, only LF phase was associated with improved outcome (Table 2; adjusted: OR=5.37, 95% CI:1.31-22.0, p=0.023). Results for ordinal mRS are shown in Table S6, Figures S2,3.

##### Unaffected hemisphere

There was no association between dCA variables from the unaffected hemisphere and mRS at any time point. These results are reported in Table S7.

##### dCA parameters over time

dCA parameters are summarized at each time point in Table 3. Differences across time points were observed for CBv (F_(3,447)_=9.7, p<0.001), VLF phase (F_(3,448)_=6.8, p<0.001), VLF gain (F_(3,146.7)_=45.8, p<0.001), LF phase (F_(3,140.1)_=9.8, p<0.001), and LF gain (F_(3,145.6)_=28.6, p<0.001). Mean CBv was greatest at 24–72h after AIS (50.6±19.9 cm/s), and lowest at 4–7d (37.4±20.1 cm/s), compared to values <24h (46.7±18.6 cm/s) and at 3mo (41.4±15.7 cm/s). VLF phase was highest at 4-7d (1.02±0.45rad), but similar at other time points. LF phase was highest (0.87±0.56rad) <24h of stroke, and falling steadily across timepoints, being lowest at 3mo (0.6±0.48rad).

##### Covariates, dCa parameters and outcome (mRS)

All analyses are presented in Tables S8,9. On univariable analysis, higher initial NIHSS was associated with poor outcome (mRS>2) for all groups at all time points. Older age was associated with poor outcome at all time points except 4-7 days. Non-lacunar was associated with poor outcome only within 24h. Diabetes and atrial fibrillation were associated with poor outcome within 72h. Stroke severity was associated with VLF phase and ARI (<24h), CBv and ARI (24-72h), VLF phase and LF gain (4-7d), but no associations were seen at 3mo.

## Discussion

Using a one-stage IPDMA, we found that dCA in the affected hemisphere within 72h post-AIS is prognostic for outcome (mRS) at 3mo. Furthermore, dCA in the affected hemisphere (VLF phase), has a major oscillation in the acute and subacute phases with a slight decrease, followed by an increase 4-7d post-AIS.

Importantly, dCA in the affected hemisphere, in the acute stage post-AIS, had a significant association with functional outcome. In particular, higher VLF phase <24h was prognostic of improved functional outcome (80% reduction in the probability of having mRS>2 at 3mo). Thus, disturbed dCA at the earliest stage post-AIS, was most prognostic for long-term functional outcome. However, measurements across multiple time points will identify those with stable, recovering, or deteriorating dCA, and may help prognosticate long-term outcomes. This finding reinforces those of previous studies in more severe AIS^2,24,25^. During the acute stage, intact dCA could restore the penumbral area via buffering of BP oscillations through resistance vessels of the reperfused area, avoiding further ischemic damage, edema and hemorrhagic transformation^1,16,25^. Taken together, these findings support the concept that dCA should be used to guide individualized BP management, demonstrated in a recent observational study^24^.

The role of dCA in AIS has been extensively studied. Although there is little evidence of compromised dCA after the first week of AIS^20^, robust evidence exists that dCA is impaired in the affected hemisphere at <72h^1^, extending to the subacute stage (1 week)^26^, and is associated with stroke severity^2^. Moreover, dCA may also be altered in the unaffected hemisphere, in the subacute phase^6,20,27^.

In terms of temporal changes, there was an oscillation of dCA in the affected hemisphere in the first week, demonstrating that cerebral blood flow is more susceptible to fluctuations in BP at this stage in line with previous publications^1,20,26^. Contrary to previous research^6,20,27^, we did not find any difference between time points in the unaffected hemisphere. This may be due to the larger sample size afforded by the IPDMA, with correction for confounding factors. Secondly, the population studied in this IPDMA predominantly comprised large vessel occlusion stroke, which maybe more likely to induce unilateral changes in dCA in the absence of chronic small vessel disease^1^. However, including patients with lacunar infarct was meaningful, given that dCA disruption from acute lacunar infarction, in addition to pre-existing dCA impairment from chronic small vessel disease, may result in particularly challenging BP management for these patients. Additional findings suggest ongoing modification of LF phase that extends to the chronic stage but, as dCA is frequency-dependent and all the studies used spontaneous oscillations in BP and CBv, we considered the VLF phase more representative of the pathophysiological mechanisms involved in cerebrovascular regulatory dysfunction during AIS^28^. In addition, the oscillation of dCA in the acute stage has been demonstrated to have a clear correlation with response to reperfusion therapies, reinforcing the theory that dCA may be linked to the size of ischemic lesion^6,19,29^.

This is the first, multi-center IPDMA of the association between dCA and functional outcome after AIS. However, our study has several limitations, which need to be further discussed: 1) the populations gathered were heterogeneous with different patients evaluated at different time points; making inferences on the temporal evolution of dCA difficult to ascertain; 2) demographic and clinical parameters, especially at 4–7d, were significantly different from other time points; and dCA metrics at 4–7d and 3mo came from only two centers; 3) sample sizes were limited for some analyses due to missing outcome, dCA or covariate data; 4) we identified several centers from whom we did not manage to obtain data for this IPDMA, impacting the generalizability of the findings; 5) we were not able to conduct all of the prespecified analyses; deviations from the pre-published protocol are available in SM2; 6) only mRS at 3mo had sufficient data for analysis and we were not able to analyze other outcomes (e.g. NIHSS, infarct volume/extent); 7) phase wrap around effect cannot be fully excluded, which would require re-analysis of the raw data.

Future work should: 1) follow the CARNet White Paper criteria to reduce heterogeneity in data collection and reporting; 2) investigate the relationship between sex, ethnicity, and dCA parameters following AIS; and 3) metaregression should be used to explore the impact of HR, BP, and EtCO_2_ on dCA and outcome following AIS. The findings presented herein support the development of a future, multi-center clinical trial to investigate the role of dCA monitoring to guide treatments and interventions, particularly during the early phase post-AIS (<24h, 24-72h), with a range of clinical outcomes in different stroke subtypes (lacunar versus non-lacunar), in the medium (3-6mo) and longer-term (>12mo).

## Conclusions

This large IPDMA shows that dCA metrics collected early after AIS are prognostic of functional outcome at three months, and that dCA has considerable time-varying behavior. These findings will guide further prospective randomized multi-center trials to optimize treatment in AIS based on dCA assessments.

## Supplementary Material

Graphical Abstract

Supplemental Publication Material clean

## Figures and Tables

**Table 1:** Summary of primary studies from the index publications from which data were derived. AIS=acute ischemic stroke, ARI=autoregulation index, BP=blood pressure, CAI=cerebral augmentation index, dCA=dynamic cerebral autoregulation, EtCO_2_=end-tidal carbon dioxide, d=days, h=hours, HR=heart rate, MCA=middle cerebral artery, mo=months, mRS=modified Rankin Scale, NIHSS=National Institute for Health Stroke Scale, NR= not reported, STROBE=Strengthening the Reporting of Observational Studies in Epidemiology. Age is given as mean ± SD or range (min-max).

Study ID	Quality score (STROBE)	Country (center)	Sample size	Age (years)	Sex (M:F)	Population	Time point measures conducted	dCA variables	BP, EtCO_2_, and HR monitoring	Outcomes and follow-up duration
Atkins, 2010^15^	17	UK (3)	19	67 ± 11	9:10	Mild AIS (NIHSS<8) within 48h of onset	24–48h, 96h	ARI	BP, HR	NIHSS, 96 h, (mRS 3mo unpublished)
Castro, 2017a^16^	17	Portugal (1)	46	73 ± 12	25:21	AIS: MCA territory	Within 6h, at 24h, and 3mo	Phase, gain, coherence	BP, HR, EtCO_2_	Hemorrhagic transformation, cerebral edema, 24h, (mRS 3mo unpublished)
Castro, 2017b^17^	16	Portugal (1)	30	69 ± 13	16:14	AIS: MCA territory	Within 6h	Phase, gain, coherence	BP, HR, EtCO_2_	mRS, 3mo
Chi, 2018^18^	16	Taiwan (6)	86	57–59	66:20	AIS with premorbid mRS of 0	3-7d	Phase, gain, coherence	BP	mRS at 3mo
Lam, 2019^23^	18	UK (3)	15	69 ± 7.5	8:7	AIS within 24h	Within 24h, 7d, 3mo	ARI	BP, HR, EtCO_2_	mRS and NIHSS, 7d and 3mo
Nogueira, 2020^19^	17	UK, Brazil (3,4)	38	65–68	NR	AIS eligible for thrombolysis (within 4.5h of onset)	During thrombolysis and 247#x2013;48h after treatment	Phase, gain, coherence, ARI	BP, HR, EtCO_2_	NIHSS at end of therapy and 24–48h (mRS 3mo unpublished)
Panerai, 2016^21^	17	UK (3)	11	62 (39–87)	8:3	AIS within 48 h of onset	Within 72h	ARI	BP, HR, EtCO_2_	NR (mRS 3mo unpublished)
Saeed, 2013^22^	16	UK (3)	22	60-65	14:8	AIS within 48h of onset	Within 48h	ARI	BP, HR, EtCO_2_	NR (mRS 3mo unpublished)
Salinet, 2014^20^	18	UK (3)	15	62 ± 9	12:3	AIS within 72h of onset	Within 72h, 14d, 1 and 3mo	ARI	BP, HR, EtCO_2_	NR (mRS 3mo unpublished)
Salinet, 2019^2^	20	Brazil (4)	55	62–63	28:27	MCA territory AIS (mild, moderate, and severe) within 48h of onset	Within 48h	ARI	BP, HR, EtCO_2_	mRS, deaths, vascular events at 3mo
Xiong, 2015^11^	14	China (6)	72	63 ± 10	63:9	AIS within 7 days of onset	Within 7d	CAI	BP	mRS, 6mo (3mo unpublished)

**Table 2:** Univariable analyses for dCA parameters from the affected hemisphere for modified Rankin Scale (mRS) as good [mRS:0–2] vs. poor [mRS:3–6] outcome. Analyses were conducted with generalized linear mixed models with the center of origin included as a random effect; adjusted for: stroke severity, age, stroke sub-type, diabetes, and antihypertensives (<24h, 24-72h), only stroke severity and age (4d, 3mo, ARI and Coherence). P_FDR_=p-value corrected for multiple comparisons with false discovery rate approach, AdjOR= adjusted OR, ARI=autoregulation index, CBv=cerebral blood velocity, d=days, h=hours, LF=low frequency, mo=months, OR=odds ratio, SE=standard error, VLF=very low frequency. Phase measured in radians. Measures with means < 1 (VLF Phase, VLF Gain, FL Phase, LF Gain, Coherence) were rescaled to represent changes per 1 SD. **Bolded** values indicate uncorrected P < 0.05.

	n	Beta	SE	P	P_FDR_	OR	L95%	U95%	AdjOR
**<24h**									
**CBv**	189	-0.005	0.009	0.56	0.672	1.01	0.99	1.02	1.00
**VLF Phase**	**190**	**-0.774**	**0.197**	**<0.001**	**<0.001**	**2.17**	**1.47**	**2.64**	**2.29**
**VLF Gain**	190	-0.001	0.167	0.995	0.995	1.00	0.72	1.18	0.97
**LF Phase**	192	-0.126	0.172	0.41	0.547	1.13	0.81	1.35	**1.49**
**LF Gain**	192	-0.217	0.170	0.20	0.30	1.24	0.89	1.47	1.09
**ARI**	**49**	**–0.376**	**0.183**	**0.04**	**0.12**	**1.46**	**1.02**	**2.08**	1.48
**Coherence**	52	-0.134	0.351	0.08	0.192	1.14	0.57	2.27	1.33
**24–72h**									
**CBv**	**121**	**0.028**	**0.012**	**0.016**	**0.09**	**0.97**	**0.95**	**1.00**	0.95
**VLF Phase**	**121**	**-0.666**	**0.242**	**0.006**	**0.07**	**1.95**	**1.21**	**2.27**	**2.50**
**VLF Gain**	120	0.413	0.213	0.053	0.141	0.66	0.44	1.00	0.84
**LF Phase**	**122**	**-0.642**	**0.266**	**0.016**	**0.09**	**1.90**	**1.13**	**3.20**	**2.58**
**LF Gain**	**121**	**0.553**	**0.234**	**0.018**	**0.09**	**0.58**	**0.36**	**0.91**	0.71
**ARI**	**23**	**–0.68**	**0.299**	**0.023**	**0.09**	**0.66**	**0.53**	**0.82**	4.22
**Coherence**	24	-0.727	0.477	0.13	0.26	2.07	0.81	5.27	3.38
**4–7d**									
**CBv**	99	0.013	0.014	0.337	0.475	0.99	0.96	1.01	0.99
**VLF Phase**	99	-0.455	0.348	0.153	0.282	1.57	0.80	3.12	1.26
**VLF Gain**	99	0.394	0.285	0.116	0.253	0.67	0.39	1.18	0.93
**LF Phase**	84	0.231	0.391	0.554	0.672	0.79	0.37	1.71	0.64
**LF Gain**	84	-1.163	0.877	0.184	0.294	3.21	0.57	17.9	4.28
**ARI**	–	–	–	–	–	–	–	–	-
**Coherence**	–	–	–	–	–	–	–	–	-
**3mo**									
**CBv**	39	–0.008	0.020	0.72	0.785	1.01	0.97	1.05	0.94
**VLF Phase**	39	-0.482	0.355	0.175	0.294	1.62	0.81	3.25	1.93
**VLF Gain**	39	-0.045	0.331	0.893	0.932	1.05	0.55	2.00	1.05
**LF Phase**	**39**	**-1.109**	**0.516**	**0.032**	**0.11**	**3.03**	**1.10**	**8.33**	**5.37**
**LF Gain**	39	-0.141	0.326	0.665	0.76	1.15	0.61	2.18	2.21
**ARI**	–	–	–	–	–	–	–	–	-
**Coherence**	–	–	–	–	–	–	–	–	-

**Table 3:** Mean and standard deviation (SD) for autoregulatory parameters in the affected hemisphere. ARI= autoregulation index, CBv= cerebral blood velocity, LF= low frequency, VLF= very low frequency. P-values from comparison across time points using with ANOVA or t-test, using Welch’s correction in case unequal variances.

	Total			< 24h			24-72h			4-7d			3mo			Across time comparison
	**n**	**mean**	**SD**	**n**	**mean**	**SD**	**n**	**mean**	**SD**	**n**	**mean**	**SD**	**n**	**mean**	**SD**	**p-values**
**CBv, cm/s**	361	44.8	19.7	189	46.7	18.6	121	50.6	19.9	99	37.4	20.1	39	41.4	15.7	<0.001
**VLF** **Phase, rad**	362	0.85	0.46	190	0.80	0.44	121	0.75	0.47	99	1.02	0.45	39	0.82	0.53	<0.001
**VLF Gain cm ·s-1 · mmHg-1**	361	0.67	0.53	190	0.78	0.51	120	0.82	0.66	99	0.44	0.21	39	1.08	0.43	<0.001
**LF Phase rad**	350	0.75	0.52	192	0.87	0.56	122	0.59	0.37	84	0.65	0.53	39	0.60	0.48	0.002
**LF Gain cm ·s-1· mmHg-1**	349	0.77	0.58	192	0.80	0.52	121	1.04	0.71	84	0.58	0.42	39	1.34	0.46	<0.001
**ARI**	72	4.8	2.1	49	4.80	2.11	23	4.67	2.09	-	-	-	-	-	-	0.805
**Coherence**	76	0.59	0.24	52	0.56	0.24	24	0.66	0.25	-	-	-	-	-	-	0.102
