# Supplementary figures and images for: Individual patient data meta-analysis of dynamic cerebral autoregulation and functional outcome after ischemic stroke
Source: Stroke. Author manuscript; Available in PMC 2024 May 1. (PMC7615849; doi:10.1161/STROKEAHA.123.045700)

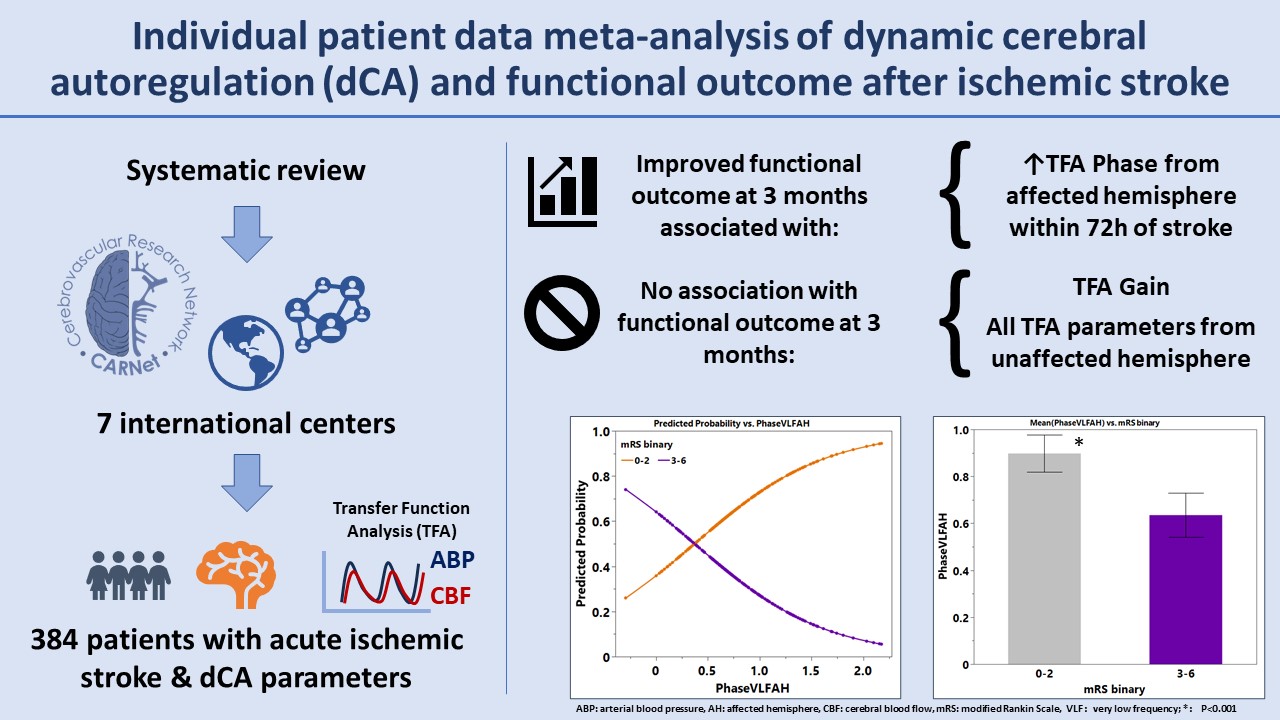

Supplement: Graphical Abstract [file EMS194373-supplement-Graphical_Abstract_.jpg]
