# Supplementary material for: Individual patient data meta-analysis of dynamic cerebral autoregulation and functional outcome after ischemic stroke
Source: Stroke. Author manuscript; Available in PMC 2024 May 1. (PMC7615849; doi:10.1161/STROKEAHA.123.045700)
Supplement: Supplemental Publication Material clean [file EMS194373-supplement-Supplemental_Publication_Material_clean_.pdf]

SUPPLEMENTAL MATERIAL

Supplementary Methods

1. Searches and screening

Embase, Medline, Web of Science, PsycInfo, and Cochrane were screened from inception to 31/03/2021 using a pre-specified research strategy. The search strategy was developed in conjunction with librarians based at the University of Leicester, and was adapted for each database searched.

In total, 3919 papers were found after removal of duplicates. Articles were screened on title and abstract by three reviewers in pairs (LB & SB, LB & YG) initially against the following inclusion criteria: Adults aged >18 years, diagnosis of AIS (all sub-types), cerebrovascular parameters available, including indices of dCA (up to 12 months post-symptom onset), and exclusion criteria: age <18 years, haemorrhagic stroke, subarachnoid haemorrhage, no measures of cerebral haemodynamics, measurements beyond 12 months only, animal studies, systematic review/meta-analysis, or conference abstract. Disagreements between reviewers were resolved by discussion.

Following title and abstract screening, 137 papers were reviewed independently at full text, or the authors were contacted where full texts were not available, by three reviewers in pairs (LB & EH, LB & PR). Disagreements were resolved by discussion. All screening was performed using Covidence©. Out of 137 papers, we contacted 83 authors to see if data were available for inclusion. We contacted authors at least twice for each relevant study/publication. We also contacted authors who published TCD studies of cerebral blood velocity (CBv) in ischaemic stroke populations to identify if dCA analyses were conducted but not published. We often identified multiple papers by the same author, using the same dataset, in this instance we asked the author to identify the publication related to the primary data collection, or the most methodological information for quality assessment. Where data were not available, or there was no response after two contacts, these studies were excluded from the IPDMA. Given this was a one-stage IPDMA and there has been a recent systematic review and meta-analysis of aggregate level study data, we did not perform an additional two-stage approach[12]. SM4 shows the modified PRISMA flowchart for reasons for exclusion based on full text screening or author contact. Although 47 authors contacted for further data did not respond, the majority of these conducted TCD-based assessments of CBv and did not undertake dCA analyses in the primary publication, and so were unlikely to have this data available for analysis.

2. Protocol modifications

The protocol for this IPDMA was modified and updated to reflect the data acquired as part of the review process. The major changes were to the data analysis, firstly as a result of fewer than anticipated centres contributing data, and secondly as a result of the heterogeneity in data available in terms of outcomes, time points, and comorbidities. We focussed this analysis on the first modelling phase outlined in the original protocol[9], with the intention to describe the changes in dCA occurring at different time points following AIS, and exploring this relationship with outcome in AIS.

3. Quality assessment and publication bias

We assessed the quality of included data based on the primary research papers from which the data were derived by comparing the study methods against the recently updated CARNet White Paper criteria[10]. The CARNet White Paper outlines best practice for the conduct of TCD-based cerebral autoregulation research, and covers domains such as data acquisition and pre-processing, transfer function methodology and reporting, alternative metrics, and normative data and thresholds[10]. Quality of study reporting for observational studies was assessed using the Strengthening the Reporting of Observational studies in Epidemiology (STROBE) guidelines[13]. Quality assessment using the STROBE criteria was conducted independently by two reviewers (LB & EH), and the assessment against the CARNet white paper criteria was conducted independently by pairs of reviewers (LB & JM, SP & DS, AS & RN, PC & MM, AR & PB). Disagreements were resolved by discussion between reviewers. Quality assessments were based on the original, published reports and were conducted independently of the IPDMA. A statistical assessment of publication bias using funnel plots was not possible due to insufficient aggregate data provided in the original published reports. Much of the outcome data obtained for this IPDMA was unpublished as well as published and therefore reduces the risk of publication bias in this analysis.

4. Figure S1. PRISMA flow diagram for selection of studies/participating centres included in this review

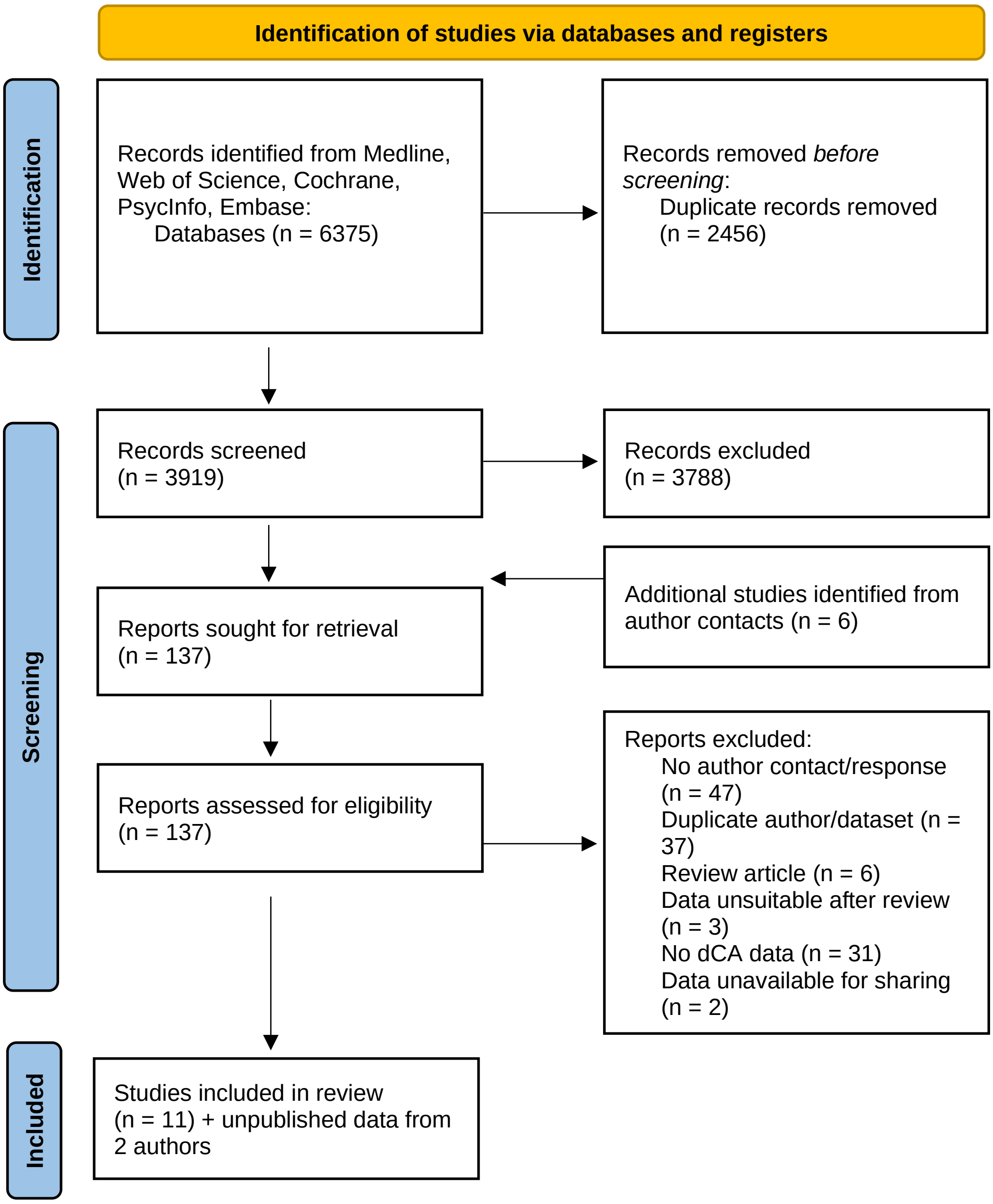

5. Search strategy

Medline 1946-date (includes epub ahead of print and in process citations. Updated daily)

1	exp Brain Ischemia/ or acute ischaemic stroke.mp.	113117
2	acute ischemic stroke.mp.	16059
3	AIS.mp.	12774
4	cerebral blood flow*.mp. or exp Cerebrovascular Circulation/	67747
5	dCA.mp.	4445
6	dynamic cerebral autoregulat*.mp.	402
7	cerebral autoregulat*.mp.	2235
8	cerebral haemodynamic*.mp.	572
9	autoregulatory index.mp.	108
10	ARI.mp.	3831
11	transfer function analysis.mp.	443
12	TFA.mp.	4114
13	(phase and gain and coherence).mp.	333
14	sit to stand.mp.	3141
15	squat stand.mp.	43
16	thigh cuff.mp.	216
17	modified rankin scale.mp.	10153
18	mRS.mp.	22019
19	death.mp. or exp Death/	898390
20	(dependent or dependence).mp.	1716620
21	exp Mortality/ or mortality.mp.	1330552
22	national institute of stroke severity scale.mp.	1
23	NIHSS.mp.	5905
24	glasgow coma scale.mp. or exp Glasgow Coma Scale/	15826
25	GCS.mp.	15995
26	barthel.mp.	6887
27	infarct size.mp.	15958
28	infarct volume.mp.	6095

29 infarct extension.mp. 150

30 infarct growth.mp. 313

31 (hemorrhagic transformation or haemorrhagic transformation).mp. 1823

32 (parenchymal haematoma or parenchymal hematoma).mp. 320

33 (cerebral oedema or cerebral edema).mp. 6970

34 17 or 18 or 19 or 20 or 21 or 22 or 23 or 24 or 25 or 26 or 27 or 28 or 29 or 30 or 31 or 32 or 33 3639258

35 4 or 5 or 6 or 7 or 8 or 9 or 10 or 11 or 12 or 13 or 14 or 15 or 16 84264

36 (ESUS or embolic stroke unknown source).mp. 350

37 (small vessel occlusion or small vessel stroke).mp. 406

38 (large vessel occlusion or LVO or large vessel stroke).mp. 2340

39 1 or 2 or 3 or 36 or 37 or 38 131321

40 34 and 35 and 39 3206

41 limit 40 to yr="2005 -Current" 1846

6. Data dictionary

Variable	Description	Variable type	Missing data	Values/labels
Study ID	Identifies the original database the data was sourced from using the principal investigator as the source	String	99=missing individual<br>999=missing study	1= Study 1<br>2= Study 2<br>3= Study 3<br>4……
Country	Country where the study took place	Categorical	N/A	
Clinical setting	Clinical setting where the study took place	Categorical	999=missing data	1= In-patient acute<br>2= In-patient rehabilitation<br>3= Out-patient
**Demographic variables**				
Age	Participant age	Continuous	99=missing data	
Sex	Participant sex	Categorical	99=missing data	0=female<br>1=male
Ethnicity	Participant ethnicity	Categorical	99=missing data	1=Caucasian<br>2=Black<br>3=Asian<br>4……
Carotid artery disease	Presence or absence of carotid artery disease as less than or greater than 50% vessel stenosis	Categorical	99=missing data	1=>50%<br>0= <50%
Diabetes	Diagnosis of diabetes (type one, two, or unknown/other type)	Categorical	99=missing data	1=present<br>0=absent
Hypertension	Diagnosis of hypertension	Categorical	99=missing data	1=present<br>0=absent
Atrial fibrillation	Diagnosis of atrial fibrillation or ECG consistent with atrial	Categorical	99=missing data	1=present<br>0=absent

	fibrillation (irregularly irregular rhythm)			
Previous stroke	History of previous stroke	Categorical	99=missing data	1=yes<br>0=no
Heart failure	Diagnosis of heart failure with or without preserved ejection fraction	Categorical	99=missing data	1=present<br>0=absent
Ischaemic heart disease	Any heart disease including: myocardial infarction (ST elevation and non-ST elevation), angina (stable and unstable), ischaemic heart disease	Categorical	99=missing data	1=present<br>0=absent
Smoking	Smoking status (tobacco including cigarettes, cigars, pipe)	Categorical	99=missing data	1=current<br>0=never<br>2=Ex-smoker
Other comorbidities	Any comorbidity not listed above	String	99=missing data	
Anti-hypertensive medication	Current treatment with anti-hypertensive medication (any class of medication)	Categorical	99=missing data	1=yes<br>0=no
Statin	Current treatment with a statin	Categorical	99=missing data	1=yes<br>0=no
Cerebrovascular disease	Presence of cerebrovascular disease on brain imaging (mild, moderate or severe)	Categorical	99=missing data	1=present<br>0=absent
Pre-morbid mRS	Pre-stroke modified Rankin scale	Ordinal	99=missing data	0=no symptoms<br>1=no significant disability<br>2=slight disability<br>3=moderate disability<br>4=moderate-severe disability<br>5=severe disability

				6=dead
Frailty	Level of frailty determined by a validated scale (e.g. clinical frailty scale). Record which scale was used.	Categorical	99=missing data	1=very fit<br>2=fit<br>3=managing well<br>4=very mild frailty<br>5=mild frailty<br>6=moderate frailty<br>7=severe frailty<br>8=very severe frailty<br>9=terminally ill
Thrombolysis	Received thrombolysis for treatment of acute stroke in hospital	Categorical	99=missing data	1=yes<br>0=no
NIHSS initial	National Institutes of Health Stroke Scale first recorded after stroke onset and time point the assessment was made at post-event	Continuous	99=missing data	
Time to randomisation	Time (minutes) from event to randomisation if the study is a clinical trial	Continuous	99=missing data	
Time to thrombolysis/thrombectomy	Time (minutes) from event to thrombolysis or thrombectomy (whichever occurred first)	Continuous	99=missing data	
**Stroke characteristics**				
Hemisphere affected	Left or right hemisphere affected by stroke and whether this was in the middle cerebral artery or the posterior artery circulation	Categorical	99=missing data	1=Right middle cerebral artery (RMCA)<br>2=Left middle cerebral artery (LMCA)<br>3=Posterior circulation (PC)<br>4=multiple

Stroke subtype	Source of the stroke was large vessel or non-large vessel in origin	Categorical	99=missing data	0=Non-large vessel occlusion (NLVO)<br>1=Large vessel occlusion (LVO)
Bamford classification	Classification of the stroke as specified by the Bamford criteria			1=PACS<br>2=LACS<br>3=TACS<br>4=POCS
CT angiography	Classification of stroke by CT angiographic imaging	Categorical	99=missing data	1=ICA<br>2=T<br>3=M1<br>4=M2<br>5=M3<br>6=P1/V4/BA
Haemorrhagic transformation	As classified by European Cooperative Acute Stroke Study (ECASS II) classification	Categorical	99=missing data	0=none<br>1=HI1<br>2=HI2<br>3=PH1<br>4=PH2
Oedema	Presence of oedema as identified on brain imaging	Categorical	99=missing data	1=present<br>0=absent
**Clinical outcomes**				
NIH final	NIHSS score recorded at follow-up post-event and the time-point at which the assessment was made	Continuous	99=missing data	
Baseline mRS	mRS recorded at stroke onset (baseline)	Ordinal	99=missing data	
Outcome mRS	mRS recorded at follow-up and at what time points assessments were made post-event	Ordinal	99=missing data	
Baseline Barthel index		Continuous	99=missing data	

Outcome Barthel index	Barthel index recorded at follow-up and the time-point at which the assessment was made	Continuous	99=missing data	
Glasgow coma scale	Glasgow come scale score at event, or the earliest recorded post-event and at follow-up	Continuous	99=missing data	
Infarct volume	Volume (mm^3) of infarcted tissue as measured on brain imaging	Continuous	99=missing data	
Infarct extension	Presence of infarct extension post-event	Categorical	99=missing data	1=present<br>0=absent
Number of infarcts/Frazekas score	Number of acute infarcts present on brain imaging and Frazekas score if available	Continuous	99=missing data	
**dCA variables***				
CBv	Cerebral blood velocity (cm/s)	Continuous	99=missing data	
Phase VLF	Phase at very low frequency (0.02-0.07 Hz)	Continuous	99=missing data	
Gain VLF	Gain at very low frequency (0.02-0.07 Hz)	Continuous	99=missing data	
Phase LF	Phase at low frequency (0.07-0.2 Hz)	Continuous	99=missing data	
Gain LF	Gain at low frequency (0.07-0.2 Hz)	Continuous	99=missing data	
ARI	Autoregulatory index (derived from transfer function analysis not thigh cuff manoeuvre)	Ordinal	99=missing data	
Coherence	Coherence of transfer function analysis	Continuous	99=missing data	
Mx	Mean flow index	Continuous	99=missing data	

Prx	Pressure reactivity index	Continuous	99=missing data	
Toxa	NIRS derived index of autoregulation	Continuous	99=missing data	
**Physiological variables**				
Arterial blood pressure	Arterial blood pressure from beat-to-beat monitoring	Continuous	99=missing data	
End-tidal CO_2	End-tidal CO_2	Continuous	99=missing data	
Heart rate	Heart rate	Continuous	99=missing data	

Data dictionary summarising the data points collected (where available) for this IPDMA, the definitions for each variable, data type, and codes assigned. *dCA parameters for the affected and unaffected hemisphere categorised by time point of measurement from event (within 24 hours, 24-72 hours, 4-7 days, and more than 3 months). ARI= autoregulatory index, CBv= cerebral blood velocity, ECASS= European Cooperative Acute Stroke Study, ECG= electrocardiogram, LACS= lacunar stroke, LF= low frequency, LVO= large vessel occlusion, MCA= middle cerebral artery, mRS= modified Rankin Scale, Mx= mean flow index, NIHSS= National Institutes of Health Stroke Scale, NIRS= near-infrared spectroscopy, PACS= partial anterior circulation stroke, PCA= posterior cerebral artery, POCS= posterior circulation stroke, Prx= pressure reactivity index, TACS= total anterior circulation stroke, VLF= very low frequency.

PRISMA-IPD Checklist

PRISMA-IPD Section/topic	Item No	Checklist item	Reported on page
**Title**			
Title	1	Identify the report as a systematic review and meta-analysis of individual participant data.	1
**Abstract**			
Structured summary	2	Provide a structured summary including as applicable:	5,6
		**Background**: state research question and main objectives, with information on participants, interventions, comparators and outcomes.	
		**Methods**: report eligibility criteria; data sources including dates of last bibliographic search or elicitation, noting that IPD were sought; methods of assessing risk of bias.	
		**Results**: provide number and type of studies and participants identified and number (%) obtained; summary effect estimates for main outcomes (benefits and harms) with confidence intervals and measures of statistical heterogeneity. Describe the direction and size of summary effects in terms meaningful to those who would put findings into practice.	
		**Discussion:** state main strengths and limitations of the evidence, general interpretation of the results and any important implications.	
		**Other:** report primary funding source, registration number and registry name for the systematic review and IPD meta-analysis.	
**Introduction**			
Rationale	3	Describe the rationale for the review in the context of what is already known.	7-10
Objectives	4	Provide an explicit statement of the questions being addressed with reference, as applicable, to participants, interventions, comparisons, outcomes and study design (PICOS). Include any hypotheses that relate to particular types of participant-level subgroups.	10
**Methods**			
Protocol and registration	5	Indicate if a protocol exists and where it can be accessed. If available, provide registration information including registration number and registry name. Provide publication details, if applicable.	11
Eligibility criteria	6	Specify inclusion and exclusion criteria including those relating to participants, interventions, comparisons, outcomes, study design and characteristics (e.g. years when conducted, required minimum follow-up). Note whether these were applied at the	11, SM1

		study or individual level i.e. whether eligible participants were included (and ineligible participants excluded) from a study that included a wider population than specified by the review inclusion criteria. The rationale for criteria should be stated.	
Identifying studies - information sources	7	Describe all methods of identifying published and unpublished studies including, as applicable: which bibliographic databases were searched with dates of coverage; details of any hand searching including of conference proceedings; use of study registers and agency or company databases; contact with the original research team and experts in the field; open adverts and surveys. Give the date of last search or elicitation.	11, SM1
Identifying studies - search	8	Present the full electronic search strategy for at least one database, including any limits used, such that it could be repeated.	SM5
Study selection processes	9	State the process for determining which studies were eligible for inclusion.	11, SM1
Data collection processes	10	Describe how IPD were requested, collected and managed, including any processes for querying and confirming data with investigators. If IPD were not sought from any eligible study, the reason for this should be stated (for each such study).	SM1, 11
		If applicable, describe how any studies for which IPD were not available were dealt with. This should include whether, how and what aggregate data were sought or extracted from study reports and publications (such as extracting data independently in duplicate) and any processes for obtaining and confirming these data with investigators.	
Data items	11	Describe how the information and variables to be collected were chosen. List and define all study level and participant level data that were sought, including baseline and follow-up information. If applicable, describe methods of standardising or translating variables within the IPD datasets to ensure common scales or measurements across studies.	11,12, SM6
IPD integrity	A1	Describe what aspects of IPD were subject to data checking (such as sequence generation, data consistency and completeness, baseline imbalance) and how this was done.	11
Risk of bias assessment in individual studies.	12	Describe methods used to assess risk of bias in the individual studies and whether this was applied separately for each outcome. If applicable, describe how findings of IPD checking were used to inform the assessment. Report if and how risk of bias assessment was used in any data synthesis.	SM3
Specification of outcomes and effect measures	13	State all treatment comparisons of interests. State all outcomes addressed and define them in detail. State whether they were pre-specified for the review and, if applicable, whether they were primary/main or secondary/additional outcomes. Give the principal measures of effect (such as risk ratio, hazard ratio, difference in means) used for each outcome.	12, 13
Synthesis methods	14	Describe the meta-analysis methods used to synthesise IPD. Specify any statistical methods and models used. Issues should include (but are not restricted to):<br>• Use of a one-stage or two-stage approach.<br>• How effect estimates were generated separately within each study and combined across studies (where applicable).<br>• Specification of one-stage models (where applicable) including how clustering of patients within studies was accounted for.	12, 13

		• Use of fixed or random effects models and any other model assumptions, such as proportional hazards.<br>• How (summary) survival curves were generated (where applicable).<br>• Methods for quantifying statistical heterogeneity (such as I^2 and τ^2).<br>• How studies providing IPD and not providing IPD were analysed together (where applicable).<br>• How missing data within the IPD were dealt with (where applicable).	
Exploration of variation in effects	A2	If applicable, describe any methods used to explore variation in effects by study or participant level characteristics (such as estimation of interactions between effect and covariates). State all participant-level characteristics that were analysed as potential effect modifiers, and whether these were pre-specified.	12, 13
Risk of bias across studies	15	Specify any assessment of risk of bias relating to the accumulated body of evidence, including any pertaining to not obtaining IPD for particular studies, outcomes or other variables.	11, 12, SM3
Additional analyses	16	Describe methods of any additional analyses, including sensitivity analyses. State which of these were pre-specified.	12, 13
**Results**			
Study selection and IPD obtained	17	Give numbers of studies screened, assessed for eligibility, and included in the systematic review with reasons for exclusions at each stage. Indicate the number of studies and participants for which IPD were sought and for which IPD were obtained. For those studies where IPD were not available, give the numbers of studies and participants for which aggregate data were available. Report reasons for non-availability of IPD. Include a flow diagram.	SM5
Study characteristics	18	For each study, present information on key study and participant characteristics (such as description of interventions, numbers of participants, demographic data, unavailability of outcomes, funding source, and if applicable duration of follow-up). Provide (main) citations for each study. Where applicable, also report similar study characteristics for any studies not providing IPD.	13-15
IPD integrity	A3	Report any important issues identified in checking IPD or state that there were none.	11
Risk of bias within studies	19	Present data on risk of bias assessments. If applicable, describe whether data checking led to the up-weighting or down-weighting of these assessments. Consider how any potential bias impacts on the robustness of meta-analysis conclusions.	16
Results of individual studies	20	For each comparison and for each main outcome (benefit or harm), for each individual study report the number of eligible participants for which data were obtained and show simple summary data for each intervention group (including, where applicable, the number of events), effect estimates and confidence intervals. These may be tabulated or included on a forest plot.	SM7
Results of syntheses	21	Present summary effects for each meta-analysis undertaken, including confidence intervals and measures of statistical heterogeneity. State whether the analysis was pre-specified, and report the numbers of studies and participants and, where applicable, the number of events on which it is based.	16-25

		When exploring variation in effects due to patient or study characteristics, present summary interaction estimates for each characteristic examined, including confidence intervals and measures of statistical heterogeneity. State whether the analysis was pre-specified. State whether any interaction is consistent across trials.<br><br>Provide a description of the direction and size of effect in terms meaningful to those who would put findings into practice.	
Risk of bias across studies	22	Present results of any assessment of risk of bias relating to the accumulated body of evidence, including any pertaining to the availability and representativeness of available studies, outcomes or other variables.	SM18
Additional analyses	23	Give results of any additional analyses (e.g. sensitivity analyses). If applicable, this should also include any analyses that incorporate aggregate data for studies that do not have IPD. If applicable, summarise the main meta-analysis results following the inclusion or exclusion of studies for which IPD were not available.	16-25
**Discussion**			
Summary of evidence	24	Summarise the main findings, including the strength of evidence for each main outcome.	26
Strengths and limitations	25	Discuss any important strengths and limitations of the evidence including the benefits of access to IPD and any limitations arising from IPD that were not available.	26, 27
Conclusions	26	Provide a general interpretation of the findings in the context of other evidence.	26, 27
Implications	A4	Consider relevance to key groups (such as policy makers, service providers and service users). Consider implications for future research.	27, 28
**Funding**			
Funding	27	Describe sources of funding and other support (such as supply of IPD), and the role in the systematic review of those providing such support.	4

A1 – A3 denote new items that are additional to standard PRISMA items. A4 has been created as a result of re-arranging content of the standard PRISMA statement to suit the way that systematic review IPD meta-analyses are reported.

© Reproduced with permission of the PRISMA IPD Group, which encourages sharing and reuse for non-commercial purpose

Table S1. Data summary

Centre	Method	Time of dCA data collection				mRS timepoint	Infarct Volume
		< 24 (n)	24-72 (n)	4 - 7 days (n)	≥ 3 months (n)		
1 - Portugal	TFA	116	50		39	3 months	yes
2 - Switzerland	TFA	40	42			3 months	yes
3 - UK	TFA / ARI	53	62			3 months	no
4 – Brazil	TFA / ARI	28	29			3 months	yes
5 – China	TFA		18	16		3 months	no
6 – Taiwan	TFA / MRx			86		3 months	yes
7 - Canada	TFA / MRx				12	not provided	yes

Summary of data provided by centres, including time points for dCA and outcome measures. ARI= Autoregulation Index, dCA= dynamic cerebral autoregulation, mRS= modified Rankin Scale, Mx= mean flow index, TFA= transfer function analysis.

Table S2. STROBE Checklist

Checklist of items that should be included in reports of observational studies[13].

	Item No	Recommendation
**Title and abstract**	1	(*a*) Indicate the study's design with a commonly used term in the title or the abstract
		(*b*) Provide in the abstract an informative and balanced summary of what was done and what was found
**Introduction**		
Background/rationale	2	Explain the scientific background and rationale for the investigation being reported
Objectives	3	State specific objectives, including any prespecified hypotheses
**Methods**		
Study design	4	Present key elements of study design early in the paper
Setting	5	Describe the setting, locations, and relevant dates, including periods of recruitment, exposure, follow-up, and data collection
Participants	6	(*a*) *Cohort study*—Give the eligibility criteria, and the sources and methods of selection of participants. Describe methods of follow-up<br>*Case-control study*—Give the eligibility criteria, and the sources and methods of case ascertainment and control selection. Give the rationale for the choice of cases and controls<br>*Cross-sectional study*—Give the eligibility criteria, and the sources and methods of selection of participants
		(*b*) *Cohort study*—For matched studies, give matching criteria and number of exposed and unexposed<br>*Case-control study*—For matched studies, give matching criteria and the number of controls per case
Variables	7	Clearly define all outcomes, exposures, predictors, potential confounders, and effect modifiers. Give diagnostic criteria, if applicable
Data sources/ measurement	8*	For each variable of interest, give sources of data and details of methods of assessment (measurement). Describe comparability of assessment methods if there is more than one group

Bias	9	Describe any efforts to address potential sources of bias
Study size	10	Explain how the study size was arrived at
Quantitative variables	11	Explain how quantitative variables were handled in the analyses. If applicable, describe which groupings were chosen and why
Statistical methods	12	(*a*) Describe all statistical methods, including those used to control for confounding
		(*b*) Describe any methods used to examine subgroups and interactions
		(*c*) Explain how missing data were addressed
		(*d*) *Cohort study*—If applicable, explain how loss to follow-up was addressed<br>*Case-control study*—If applicable, explain how matching of cases and controls was addressed<br>*Cross-sectional study*—If applicable, describe analytical methods taking account of sampling strategy
		(*e*) Describe any sensitivity analyses

Results

Participants	13*	(a) Report numbers of individuals at each stage of study—eg numbers potentially eligible, examined for eligibility, confirmed eligible, included in the study, completing follow-up, and analysed
		(b) Give reasons for non-participation at each stage
		(c) Consider use of a flow diagram
Descriptive data	14*	(a) Give characteristics of study participants (eg demographic, clinical, social) and information on exposures and potential confounders
		(b) Indicate number of participants with missing data for each variable of interest
		(c) *Cohort study*—Summarise follow-up time (eg, average and total amount)
Outcome data	15*	*Cohort study*—Report numbers of outcome events or summary measures over time
		Case-control study—Report numbers in each exposure category, or summary measures of exposure
		Cross-sectional study—Report numbers of outcome events or summary measures

Main results	16	(*a*) Give unadjusted estimates and, if applicable, confounder-adjusted estimates and their precision (eg, 95% confidence interval). Make clear which confounders were adjusted for and why they were included
		(*b*) Report category boundaries when continuous variables were categorized
		(*c*) If relevant, consider translating estimates of relative risk into absolute risk for a meaningful time period
Other analyses	17	Report other analyses done—eg analyses of subgroups and interactions, and sensitivity analyses
**Discussion**		
Key results	18	Summarise key results with reference to study objectives
Limitations	19	Discuss limitations of the study, taking into account sources of potential bias or imprecision. Discuss both direction and magnitude of any potential bias
Interpretation	20	Give a cautious overall interpretation of results considering objectives, limitations, multiplicity of analyses, results from similar studies, and other relevant evidence
Generalisability	21	Discuss the generalisability (external validity) of the study results
**Other information**		
Funding	22	Give the source of funding and the role of the funders for the present study and, if applicable, for the original study on which the present article is based

Table S3. Summary of reporting quality

Author	1	2	3	4	5	6	7	8	9	10	11	12	13	14	15	16	17	18	19	20	21	22	Total
Aries 2013	0	1	1	0	1	0	1	1	0	1	1	1	0	1	1	1	1	1	1	1	1	1	17
Atkins 2009	0	1	1	1	1	1	1	1	0	0	1	1	0	1	1	1	1	1	1	1	1	0	17
Castro 2017a	0	1	1	0	0	1	1	1	0	0	1	1	0	1	1	1	1	1	1	1	1	1	16
Castro 2017b	0	1	1	0	0	1	1	1	0	0	1	1	1	1	1	1	1	1	1	1	1	1	17
Chi 2018	0	1	1	1	0	0	1	1	0	0	1	1	0	1	1	1	1	1	1	1	1	1	16
Jia Liu 2020	0	1	1	1	1	0	1	1	0	0	1	1	0	1	1	1	1	1	1	1	1	1	17
Lam 2018	0	1	1	1	0	1	1	1	0	1	1	1	0	1	1	1	1	1	1	1	1	1	18
Nogueira 2020	0	1	1	0	0	1	1	1	1	0	1	1	0	1	1	1	1	1	1	1	1	1	17
Saeed 2016	0	1	1	1	0	1	1	1	1	0	1	1	0	1	1	1	1	1	1	1	0	1	17
Saeed 2013	0	1	1	1	0	1	1	1	1	0	1	1	0	1	1	1	1	1	0	1	0	1	16
Salinet 2019	0	1	1	1	1	1	1	1	1	0	1	1	1	1	1	1	1	1	1	1	1	1	20
Xiong 2016	0	1	1	0	0	0	0	1	0	0	1	1	0	1	1	1	1	1	1	1	1	1	14
Salinet 2014	1	1	1	1	0	1	1	1	0	0	1	1	0	1	1	1	1	1	1	1	1	1	18

Summary of reporting quality assess by the STROBE criteria. Green= compliant, red=non-compliant. Numbers correspond to the checklist copied below.

Table S4. Demographics of total sample and across each time point.

	Total Patients, n = 384		<24h, n = 211		24–72h, n = 134		4–7d, n = 99		3mo, n = 39	
	n or mean	% or SD	n or mean	% or SD	n or mean	% or SD	n or mean	% or SD	n or mean	% or SD
**Baseline NIHSS**	8.4	6.8	11.3	7.1	8.4	6.7	4.2	3.4	11.2	6.7
**Age (years)**	65.4	13.8	68.9	13.2	67.8	14.0	56.5	10.0	68.4	12.5
**Sex (female)**	123/384	33%	80/211	38%	47/134	35%	22/100	22%	18/39	46%
**Non–lacunar stroke**	235/384	61%	148/211	70%	123/157	78%	43/100	43%	34/39	87%
**Diabetes**	102/359	28%	51/198	26%	40/145	28%	41/100	41%	15/39	39%
**Arterial hypertension**	213/358	59%	100/204	49%	59/138	43%	79/100	79%	1/39	3%
**AF**	83/335	25%	74/186	40%	44/133	33%	0	0%	18/39	46%
**Smoking**	75/268	28%	35/180	19%	45/155	29%	9/17	53%	6/39	15%
**Antihypertensive s**	174/283	61%	125/203	62%	88/147	60%	9/17	53%	22/39	56%
**Statins**	148/280	53%	101/201	50%	76/146	52%	11/17	65%	16/39	41%
**BP, mmHg**	89.2	19.9	84.4	20.1	87.0	19.7	93.7	20.0	70.5	15.8
**$EtCO_2$, mmHg**	35.8	6.1	35.1	6.4	36.8	5.3	–	–	37.1	6.7
**HR, bpm**	70.9	12.8	71.4	14.3	69.6	12.7	70.8	9.4	72.7	15.6

Demographics of total sample and across each time point. Sample sizes are for patients with outcome of modified Rankin Scale at 3 months recorded. P-values from comparison across timepoints. AF=atrial fibrillation, BP=blood pressure, bpm= beat per minute, d=days, $EtCO_2$=end-tidal carbon dioxide, h=hours, HR=heart rate, mo=months, mRS=modified Rankin Scale, NIHSS=National Institute of Health Stroke Scale, SD=standard deviation.

Table S5. Modified Rankin Scores (mRS) for total sample and across each dynamic cerebral autoregulation time point

	Total, n = 384		< 24 hours, n = 211		24-72 hours, n = 134		4-7 days, n = 99		3 months, n = 39		Across time comparison
**mRS**	**n**	**%**	**n**	**%**	**n**	**%**	**n**	**%**	**n**	**%**	**p-values**
**Binary**											<0.001
0-2	268	70%	128	61%	82	61%	88	89%	22	56%	
3-6	116	30%	83	39%	54	39%	11	11%	17	44%	
**Ordinal**											<0.001
0	67	17%	32	15%	25	19%	15	15%	2	5%	
1	130	34%	50	24%	33	25%	57	58%	7	18%	
2	71	19%	46	22%	24	18%	16	16%	13	33%	
3	49	13%	27	13%	22	16%	10	10%	6	15%	
4	31	8%	21	10%	16	12%	0	0%	6	15%	
5	15	4%	14	6%	8	6%	1	1%	5	13%	
6	21	5%	21	10%	6	4%	0	0%	0	0%	

Modified Rankin Scores (mRS) collected at 3 months for total sample and across each dynamic cerebral autoregulation time point, as a binary (good 0-2 Vs poor 3-6), and ordinal outcome.

Table S6. Ordinal mRS results

	Beta	SE	P	OR	L95%	U95%
**<24h, ordinal mRS**						
**CBv**	–0.006	0.007	0.45	1.01	0.99	1.02
**Phase VLF**	**-0.399**	**0.135**	**0.003**	**1.49**	**1.14**	**1.94**
**Gain VLF**	-0.015	0.141	0.92	1.02	0.77	1.34
**Phase LF**	-0.004	0.139	0.98	1.00	0.76	1.32
**Gain LF**	-0.064	0.142	0.66	1.07	0.81	1.41
**ARI**	–0.289	0.186	0.12	1.34	0.93	1.92
**Coherence**	-0.494	0.365	0.18	1.64	0.80	3.35
**24–72h, ordinal mRS**						
**CBv**	**0.027**	**0.001**	**0.005**	**0.97**	0.97	0.98
**Phase VLF**	**-0.434**	**0.184**	**0.018**	**1.54**	**1.08**	**2.21**
**Gain VLF**	0.044	0.224	0.849	0.96	0.62	1.48
**Phase LF**	**-0.441**	**0.181**	**0.015**	**1.56**	**1.09**	**2.22**
**Gain LF**	0.378	0.209	0.072	0.69	0.45	1.03
**ARI**	–0.289	0.186	0.12	1.34	0.93	1.92
**Coherence**	-0.514	0.380	0.18	0.60	0.79	3.52

Table S6. Univariable analyses for dCA parameters from the affected hemisphere for each time point, with modified Rankin Scale (mRS) as an ordinal variable. Analyses were conducted with cumulative link mixed models with the center of origin included as a random effect. ARI=autoregulation index, CBV=cerebral blood velocity, h=hours, LF=low frequency, mo=months, mRS=modified Rankin Scale (mRS), OR=odds ratio, SE=standard error, VLF=very low frequency. Phase measured in radians. Analyses on ordinal mRS at 4-7 days and 3 months were not conducted due to insufficient sample size. Measures with means < 1 were rescaled to represent changes per 1 SD.

Figure S2. Ordinal mRS results within 24 hours

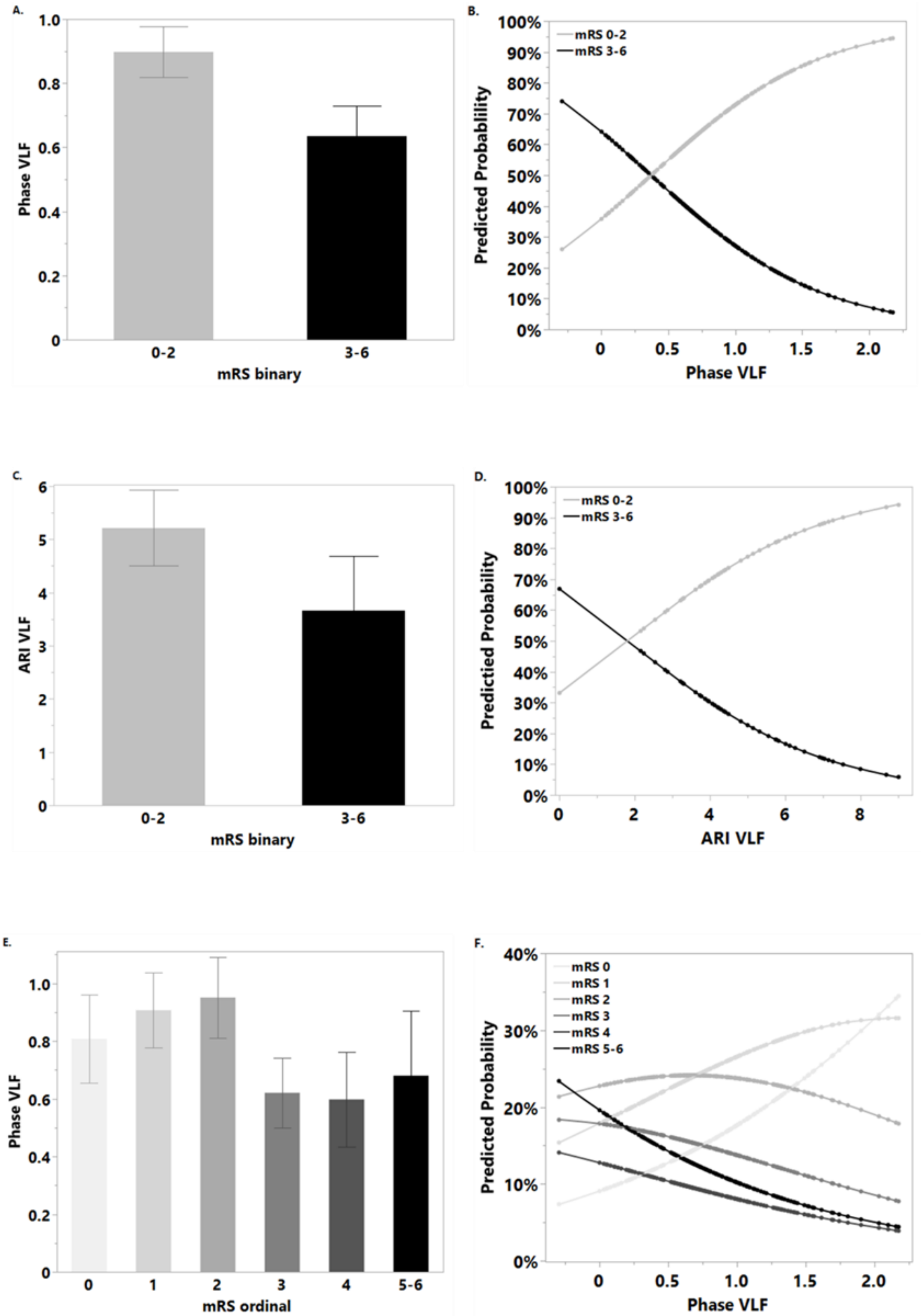

Figure S2. Mean phase at very low frequency (VLF) (A), autoregulation index (ARI) (C) in the affected hemisphere (AH) within 24h in participants with good (modified Rankin Scale [mRS]:0–2) vs poor (mRS 3–6) outcome at 3mo. The predicted probability of good vs poor outcome with increasing phase at VLF (B), and ARI (D). Mean phase VLF at all levels of the mRS (E), and the predicted probability of each mRS level with increasing phase at VLF (F).

Figure S3. Ordinal mRS results 24-72 hours

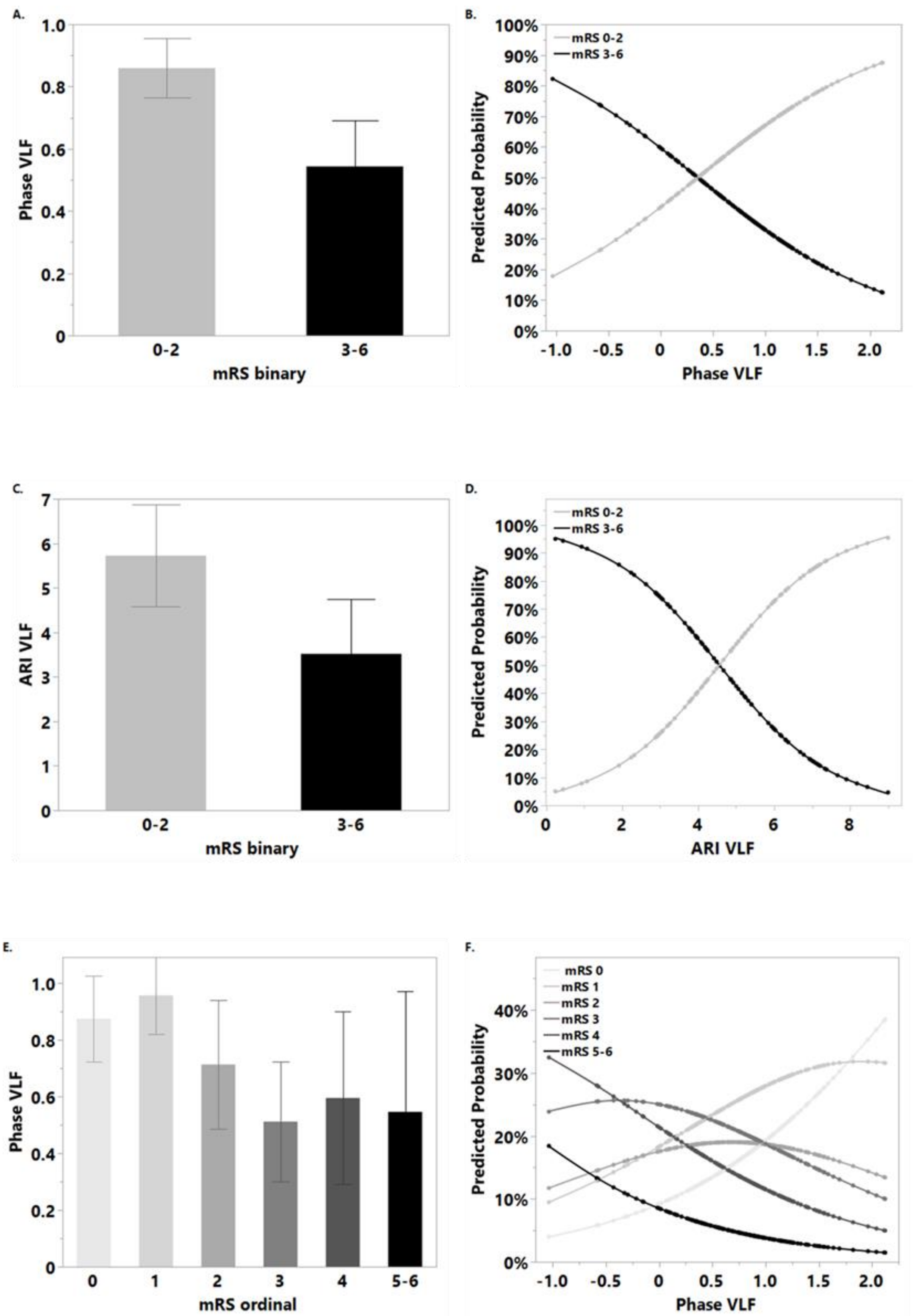

. Mean phase at very low

Figure S3. frequency (VLF) (A), autoregulation index (ARI) (C) in the affected hemisphere (AH) at 24–72h in participants with good (modified Rankin Scale [mRS]:0–2) vs poor (mRS:3–6) outcome at 3mo. The predicted probability of good vs poor outcome with increasing phase at VLF (B), and ARI (D). Mean phase VLF at all levels of the mRS (E), and the predicted probability of each mRS level with increasing phase at VLF (F)

Table S7. Univariable analyses for dCA parameters from unaffected hemisphere for each time point with raw p values reported

	Beta	SE	P	OR	L95%	U95%
**<24 hours**						
**CBv**	0.004	0.009	0.65	1.00	0.98	1.01
**Phase VLF**	-0.222	0.136	0.10	1.25	0.96	1.63
**Gain VLF**	0.096	0.159	0.55	0.91	0.67	1.24
**Phase LF**	-0.074	0.169	0.66	1.08	0.77	1.50
**Gain LF**	-0.046	0.157	0.77	1.05	0.77	1.42
**ARI**	-0.224	0.172	0.19	1.25	0.89	1.75
**Coherence**	-0.379	0.399	0.34	1.46	0.67	3.19
**24-72 hours**						
**CBv**	0.02	0.011	0.054	0.98	0.96	1.00
**Phase VLF**	-0.268	0.175	0.127	1.31	0.93	1.84
**Gain VLF**	0.215	0.205	0.295	0.81	0.54	1.21
**Phase LF**	-0.462	0.251	0.066	1.59	0.97	2.60
**Gain LF**	0.491	0.272	0.071	0.61	0.36	1.04
**ARI**	-0.515	0.292	0.078	1.67	0.94	2.97
**Coherence**	-0.600	0.563	0.286	1.82	0.60	5.49
**4-7 days**						
**CBv**	0.020	0.011	0.054	0.98	0.96	1.00
**Phase VLF**	-0.257	0.168	0.127	1.29	0.93	1.80
**Gain VLF**	0.068	0.065	0.295	0.93	0.82	1.06
**Phase LF**	-0.662	0.360	0.066	1.94	0.96	3.93
**Gain LF**	0.291	0.161	0.071	0.75	0.55	1.02
**ARI**	-	-	-	-	-	-
**Coherence**	-	-	-	-	-	-
**3 months**						
**CBv**	-	-	-	-	-	-
**Phase VLF**	-	-	-	-	-	-
**Gain VLF**	-	-	-	-	-	-
**Phase LF**	-	-	-	-	-	-
**Gain LF**	-	-	-	-	-	-
**ARI**	-	-	-	-	-	-

**Coherence**	-	-	-	-	-	-

Univariable analyses for dCA parameters from unaffected hemisphere for each time point, with mRS as binary outcome (good 0-2 Vs poor 3-6). Analyses conducted with generalised linear mixed models. Abbreviations: ARI=autoregulatory index, CBv=cerebral blood velocity, LF=low frequency, OR=odds ratio, VLF=very low frequency. Measures with means < 1 were rescaled to represent changes per 1 SD.

Table S8. Univariable analyses for covariates and outcome for each time point

	Beta	SE	P	OR	L95%	U95%
**<24 hours**						
**NIHSS initial**	0.175	0.027	**<0.001**	0.84	0.80	0.88
**Age**	0.065	0.015	**<0.001**	0.94	0.91	0.97
**Sex (Male)**	-0.059	0.299	0.84	1.06	0.59	1.90
**Non-Lacunar Stroke**	0.963	0.403	**0.017**	0.38	0.17	0.84
**Diabetes**	0.656	0.333	**0.049**	0.52	0.27	0.99
**Hypertension**	0.224	0.303	0.46	0.80	0.44	1.45
**AF**	0.75	0.306	**0.014**	0.47	0.26	0.86
**Smoking**	-0.779	0.455	0.09	2.18	0.89	5.32
**Anti-HTN Medication**	0.533	0.307	0.08	0.59	0.32	1.07
**Statins**	0.421	0.305	0.17	0.66	0.36	1.19
**ABP**	0.012	0.009	0.15	0.99	0.97	1.01
**$EtCO_2$**	-0.004	0.024	0.852	1.00	0.96	1.05
**Heart Rate**	0.004	0.011	0.72	1.00	0.97	1.02
**24-72 hours**						
**NIHSS initial**	0.244	0.049	**<0.001**	0.78	0.71	0.86
**Age**	0.058	0.019	**0.002**	0.94	0.90	0.98
**Sex (Male)**	-0.674	0.417	0.106	1.96	0.87	4.44
**Non-Lacunar Stroke**	0.65	0.547	0.234	0.52	0.18	1.52
**Diabetes**	0.18	0.454	0.692	0.84	0.34	2.03
**Hypertension**	-1.176	0.605	0.771	3.24	0.99	10.64
**AF**	1.747	0.562	**0.002**	0.17	0.06	0.52
**Smoking**	-0.649	0.478	0.175	1.91	0.75	4.88
**Anti-HTN Medication**	0.782	0.441	0.077	0.46	0.19	1.09
**Statins**	0.442	0.426	0.3	0.64	0.28	1.48
**ABP**	0.001	0.012	0.954	1.00	0.98	1.02
**$EtCO_2$**	-0.069	0.039	0.079	1.07	0.99	1.16

**Heart Rate**	-0.014	0.018	0.453	1.01	0.98	1.05
**4-7 days**						
**NIHSS initial**	0.31	0.111	**0.005**	0.73	0.59	0.91
**Age**	0.051	0.037	0.174	0.95	0.88	1.02
**Sex (Male)**	-0.674	0.417	0.106	1.96	0.87	4.44
**Non-Lacunar Stroke**	0.975	0.664	0.142	0.38	0.10	1.39
**Diabetes**	-0.75	0.719	0.297	2.12	0.52	8.70
**Hypertension**	-	-	-	-	-	-
**AF**	-	-	-	-	-	-
**Smoking**	-	-	-	-	-	-
**Anti-HTN Medication**	-	-	-	-	-	-
**Statins**	-	-	-	-	-	-
**ABP**	-0.003	0.018	0.851	1.00	0.97	1.04
**$EtCO_2$**	-	-	-	-	-	-
**Heart Rate**	-0.062	0.038	0.107	1.06	0.99	1.15
**3 months**						
**NIHSS initial**	0.284	0.099	**0.004**	0.75	0.62	0.91
**Age**	0.151	0.053	**0.004**	0.86	0.78	0.95
**Sex (Male)**	-0.486	0.653	0.46	1.63	0.45	5.85
**Non-Lacunar Stroke**	1.269	1.17	0.28	0.28	0.03	2.79
**Diabetes**	1.099	0.682	0.11	0.33	0.09	2.17
**Hypertension**	-	-	-	-	-	-
**AF**	-	-	-	-	-	-
**Smoking**	-0.511	0.934	0.58	1.67	0.27	10.42
**Anti-HTN Medication**	1.058	0.683	0.12	0.35	0.09	1.32
**Statins**	1.338	0.687	0.052	0.26	0.07	1.01
**ABP**	-0.029	0.022	0.20	1.03	0.99	1.08
**$EtCO_2$**	-0.109	0.057	0.067	1.11	0.99	1.25
**Heart Rate**	0.025	0.022	0.25	0.98	0.93	1.02

Univariable analyses for covariates for each time point, with mRS as binary outcome (good 0-2 Vs poor 3-6). Analyses conducted with generalised linear mixed models. Abbreviations: AF= atrial fibrillation, $EtCO_2$= end-tidal CO_2, NIHSS= National Institute of Health Stroke Scale,

OR=odds ratio, SE=standard error. Analyses were conducted with generalized linear mixed models.

Table S9. Stroke severity and association with dCA parameters at all time points

	Beta	SE	P
**<24 hours, NIHSS**			
**CBv**	–0.002	0.030	0.56
**VLF Phase**	**–2.359**	**1.10**	**0.032**
**VLF Gain**	–0.359	1.161	0.758
**LF Phase**	1.060	0.896	0.239
**LF Gain**	–1.067	1.148	0.354
**ARI**	**–0.942**	**0.395**	**0.022**
**Coherence**	**-10.956**	**3.742**	**0.006**
**24–72 hours, NIHSS**			
**CBv**	**0.074**	**0.012**	**0.006**
**VLF Phase**	-0.733	1.14	0.522
**VLF Gain**	0.958	1.012	0.346
**LF Phase**	-0.388	1.477	0.793
**LF Gain**	0.776	0.922	0.402
**ARI**	**-1.304**	**0.585**	**0.048**
**Coherence**	–5.844	5.034	0.270
**4–7 days, NIHSS**			
**CBv**	0.021	0.016	0.190
**VLF Phase**	**-1.947**	**0.675**	**0.005**
**VLF Gain**	3.491	2.753	0.272
**LF Phase**	-0.513	0.686	0.457
**LF Gain**	**2.424**	**0.692**	**0.001**
**ARI**	–	–	–
**Coherence**	–	–	–
**3 months, NIHSS**			
**CBv**	–0.112	0.067	0.076
**VLF Phase**	–0.171	2.084	0.935
**VLF Gain**	3.593	2.510	0.161
**LF Phase**	-0.903	2.296	0.696
**LF Gain**	2.999	2.320	0.204
**ARI**	–	–	–
**Coherence**	–	–	–

Univariable variable analyses for dCA parameters from the affected hemisphere for each time point, with NIHSS. Analyses were conducted with general linear mixed models with the center of origin included as a random effect. ARI=autoregulation index, CBv=cerebral blood velocity, d=days, h=hours, LF=low frequency, mo=months, mRS=modified Rankin Scale, OR=odds ratio, SE=standard error, VLF=very low frequency. Phase measured in radians.
